# Advances in molecular and genetic profiling of meningiomas for improved diagnosis, prognosis, and targeted therapy

**DOI:** 10.3389/fonc.2026.1750576

**Published:** 2026-03-03

**Authors:** Muthiah Kasi

**Affiliations:** Department of Radiation Oncology, PSG Institute of Oncology, PSG Institute of Medical Sciences and Research, Coimbatore, Tamil Nadu, India

**Keywords:** meningioma, molecular profiling, DNA methylation, targeted therapy, liquid biopsy, neuro-oncology, prognostic markers

## Abstract

**Background:**

Meningiomas exhibit substantial biological and clinical heterogeneity, and traditional histopathology alone often fails to accurately predict tumor behavior. Advances in molecular and genetic profiling have significantly enhanced diagnostic precision, prognostic assessment, and therapeutic decision-making.

**Methods:**

This review synthesizes current evidence on genomic, epigenomic, transcriptomic, and liquid biopsy–based approaches used to characterize meningiomas.

**Results:**

Key developments include characterization of NF2 and non-NF2 driver mutations, refinement of DNA methylation-based classification systems, identification of high-risk markers such as TERT promoter mutations and CDKN2A/B deletions, and the emergence of targeted therapeutic strategies. Liquid biopsy and circulating biomarkers further enable non-invasive disease monitoring and molecular risk stratification.

**Conclusion:**

Molecular profiling has transformed meningioma classification and risk prediction, supporting a shift toward precision neuro-oncology. Future progress will depend on integrated multi-omic diagnostics, improved biomarker-guided surveillance, and development of targeted therapeutic options for aggressive molecular subgroups.

## 1 Introduction

Meningiomas account for approximately 37% of all primary central nervous system tumors, and their incidence continues to rise, likely influenced by increased imaging use and the aging global population (1). Although most meningiomas are classified as World Health Organization (WHO) grade 1 and are generally benign, a significant proportion—estimated at 20% to 30%—display atypical or anaplastic features corresponding to grade 2 and grade 3 disease, respectively (2). These higher-grade tumors exhibit more aggressive clinical behavior, including an increased propensity for recurrence, local progression, and, in rare cases, metastatic spread. Traditionally, clinicians have relied on histopathological grading as the primary method of predicting meningioma behavior. However, considerable biological heterogeneity exists even within individual histologic grades, and several studies have demonstrated that histology alone does not reliably distinguish indolent tumors from those with high recurrence risk, even when gross total resection is achieved (3).

The past decade has witnessed a paradigm shift in meningioma research and clinical management, driven largely by the integration of molecular techniques such as next-generation sequencing, DNA methylation profiling, copy-number assessment, and transcriptional analysis. These tools have enabled investigators to uncover the genomic and epigenomic underpinnings of meningioma biology, revealing previously unrecognized tumor subgroups and refining prognostic assessments. Moreover, the development of liquid biopsy techniques and multi-omic classifiers offers promising opportunities for non-invasive tumor characterization and real-time disease monitoring. Collectively, these advances represent a significant evolution in precision neuro-oncology. This review provides a comprehensive overview of the molecular and genetic developments that have transformed diagnostic accuracy, prognostic assessment, and targeted therapeutic strategies in meningioma.

## 2 Somatic driver mutations and molecular pathogenesis

### 2.1 NF2 alterations and chromosomal instability

**Figure 1:**
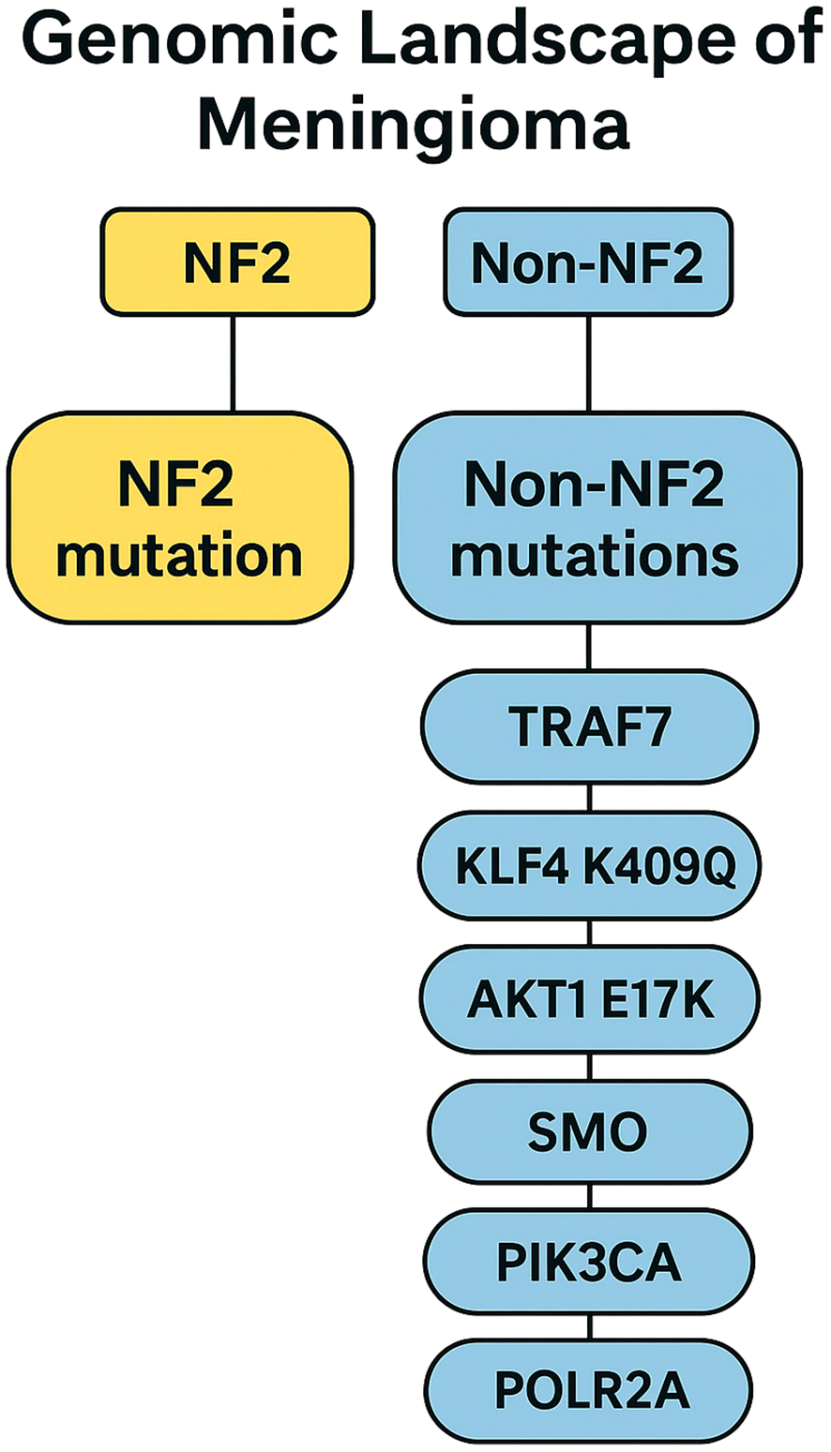
Genomic landscape of meningioma showing NF2-mutant and non-NF2 molecular subgroups and their major driver mutations.

The earliest and most extensively studied genetic event in meningioma pathogenesis involves alterations in NF2, located on chromosome 22q (**Figure 1** illustrates the genomic driver landscape, distinguishing NF2-mutant and NF2-independent molecular pathways). NF2 mutations or deletions occur in approximately 40% to 60% of sporadic meningiomas and represent the dominant driver alteration in this disease (4, 5). The NF2 gene encodes Merlin, a tumor suppressor protein that regulates contact inhibition and cytoskeletal organization. Loss of Merlin function disrupts these regulatory processes and leads to activation of multiple oncogenic pathways, including Hippo and focal adhesion kinase (FAK) signaling, promoting tumor growth and proliferation.

Clinically, NF2-mutant meningiomas tend to arise along the convexities or parasagittal regions of the brain. They frequently exhibit widespread chromosomal instability, often characterized by monosomy 22 as well as losses in chromosomes 1p, 6q, 10q, 14q, and 18q (6). These copy-number alterations contribute substantially to the aggressive potential of NF2-mutant tumors, with high copy-number burden correlating strongly with recurrence and poor outcomes.

### 2.2 Non-NF2 molecular subgroups

Advances in sequencing technologies have revealed a diverse array of NF2-independent genetic alterations that define biologically and clinically distinct molecular subgroups of meningiomas. Rather than constituting a single homogeneous category, these non-NF2 alterations encompass genes with diverse molecular functions and are associated with preferential anatomical locations, characteristic histologic subtypes, and differing prognostic implications. In general, these mutations are mutually exclusive with NF2 loss and are particularly enriched in skull-base meningiomas, which often display more stable genomic profiles and indolent clinical behavior.

Mutations in TRAF7, an E3 ubiquitin ligase, occur in approximately one-quarter of all meningiomas (5) (**Figure 1**). TRAF7-mutant tumors frequently co-occur with the KLF4 K409Q mutation, a combination that is nearly pathognomonic for secretory meningioma (5), a distinct histologic subtype characterized by eosinophilic pseudopsammoma bodies. These TRAF7/KLF4-mutant tumors typically arise from the anterior skull base, are most often classified as WHO grade 1, and generally demonstrate a benign clinical course.

Alterations in the PI3K/AKT/mTOR signaling pathway represent another major NF2-independent subgroup. Mutations in AKT1, particularly the E17K variant, result in constitutive pathway activation and are most commonly observed in midline skull-base meningiomas, including those of the planum sphenoidale and olfactory groove (7). Although these tumors are frequently WHO grade 1 by histology, they exhibit distinct radiographic and biological characteristics and have emerged as rational candidates for targeted pathway inhibition. Similarly, PIK3CA mutations occur in approximately 7% of meningiomas and are enriched in non-NF2 skull-base tumors (7). These alterations further support the biological distinction of this subgroup and provide a therapeutic rationale for PI3K or mTOR inhibition in selected patients.

Activating mutations in SMO, a key component of the Hedgehog signaling pathway, are identified in approximately 5% of meningiomas and show a strong predilection for the olfactory groove and anterior midline skull base (7). SMO-mutant tumors form a molecularly defined subgroup with characteristic transcriptional profiles and represent one of the clearest examples of a genotype-directed therapeutic opportunity in meningioma.

Beyond skull-base–predominant alterations, several additional NF2-independent mutations are associated with specific histopathologic phenotypes and prognostic implications. POLR2A mutations define a transcriptionally unique subset of largely benign meningiomas with low recurrence risk (8). SUFU mutations, though rare, implicate upstream dysregulation of Hedgehog signaling and may have implications for pathway-directed therapies (9). In contrast, loss of BAP1 function is strongly associated with rhabdoid meningioma, a high-grade variant characterized by aggressive clinical behavior and poor prognosis (10). Germline or somatic loss of SMARCE1 is characteristic of clear cell meningioma, a subtype frequently occurring in younger patients and associated with a higher risk of recurrence, particularly in spinal locations (11). Finally, mutations in ARID1A highlight the role of SWI/SNF chromatin-remodeling dysfunction in a subset of biologically aggressive and higher-grade meningiomas (12).

Together, these observations underscore that NF2-independent meningiomas represent a heterogeneous collection of molecularly, anatomically, and clinically distinct entities, rather than a single category. Recognition of this heterogeneity allows refinement of meningioma classification beyond traditional histopathology, improves prognostic stratification, and facilitates the development of personalized, genotype-informed therapeutic strategies (**Table 1** integrates driver mutations with clinical, anatomical, and prognostic associations, serving as a translational reference.).

**Table 1:** Driver Mutations and Clinical Associations.

Driver mutation	**Genomic effect/pathway**	**Clinical association**	**Typical location**	**Histologic correlation**	**Prognostic notes**
NF2	Loss of Merlin → activation of FAK, Hippo pathway dysregulation	Chromosomal instability; aggressive behavior	Convexity, parasagittal, falx	Fibrous, transitional meningiomas	High CNV burden correlates with recurrence; seen in higher-grade tumors
TRAF7	E3 ubiquitin ligase mutation	Anterior skull base meningiomas	Anterior/medial skull base	Often combined with KLF4 K409Q (secretory subtype)	Generally benign; rarely progresses
AKT1 (E17K)	Activates PI3K/AKT/mTOR pathway	Midline skull base meningiomas	Planum sphenoidale, olfactory groove	Meningothelial WHO grade 1	Potential target for AKT inhibitors; stable behavior
KLF4 (K409Q)	Transcription factor alteration	Secretory meningioma	Skull base	Secretory subtype with pseudopsammoma bodies	Indolent behavior; consistent molecular signature
PIK3CA	Activates PI3K pathway	Subset of non-NF2 skull-base tumors	Skull base	Meningothelial	Targetable with PI3K inhibitors
SMO	Hedgehog pathway activation	Olfactory groove tumors	Anterior midline base	Often meningothelial	May respond to SMO inhibitors (vismodegib)
POLR2A	RNA polymerase II subunit mutation	Distinct benign molecular class	Variable	Benign transcriptional subtype	Usually low recurrence
BAP1	Loss-of-function; chromatin remodeling defect	Rhabdoid meningiomas	Variable	Rhabdoid morphology	High-grade; very poor prognosis
SMARCE1	SWI/SNF chromatin remodeling	Clear cell meningioma (often hereditary)	Spinal canal predominance	Clear cell subtype	High recurrence risk
SUFU	Hedgehog pathway regulator	Rare SUFU-related subgroup	Variable	Uncommon	May affect Hedgehog-directed therapy
ARID1A	SWI/SNF complex alteration	Aggressive meningioma subset	Variable	High-grade histology	Associated with progression

## 3 Epigenetic profiling and DNA methylation classification

### 3.1 Development of methylation-based classification

**Figure 2:**
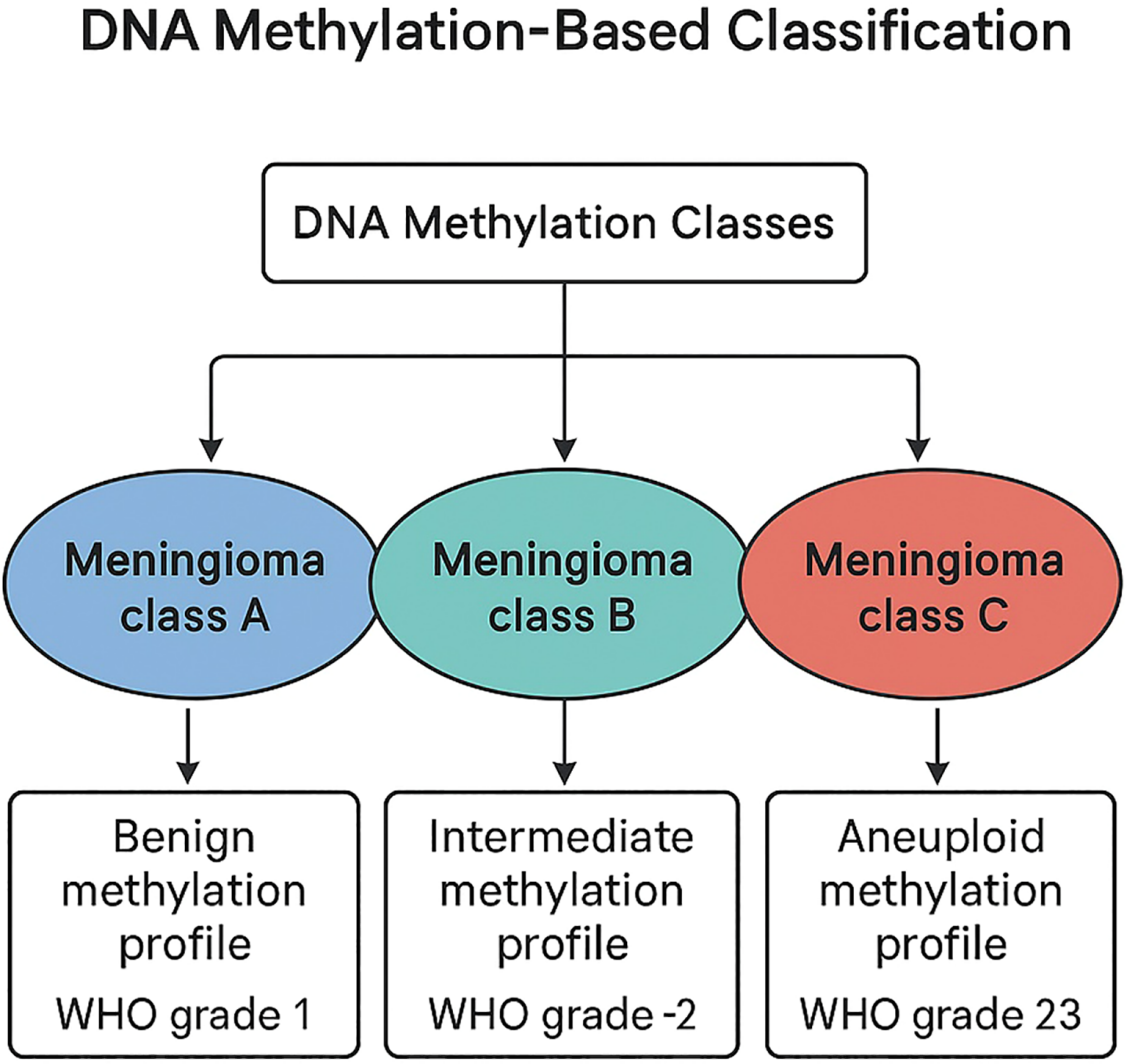
DNA methylation–based classification of meningiomas demonstrating biologically distinct classes correlated with WHO grade and prognosis.

One of the most transformative advances in meningioma research has been the incorporation of DNA methylation profiling into tumor classification (**Figure 2** depicts DNA methylation-based classification and its correlation with WHO grading and prognosis). Sahm et al. (13) pioneered a methylation-based grading system that stratifies meningiomas into biologically distinct classes with far superior prognostic accuracy compared to WHO histologic grade alone. This approach integrates genome-wide methylation signatures, copy-number patterns, and transcriptional programs to identify tumors with similar biological behaviors, regardless of histologic appearance.

### 3.2 Consensus molecular groups

Further refinement occurred through the work of Nassiri et al. (14), who conducted a large multi-institutional analysis combining methylation, transcriptomic, copy-number, and mutational data. Their study identified three major consensus molecular groups (CMGs) (**Figure 2**): a Merlin-intact group associated with favorable outcomes, an immune-enriched group characterized by inflammatory microenvironments and intermediate prognosis, and a proliferative group marked by high cell-cycle activity and poor clinical outcomes. These CMGs predict progression-free survival with greater accuracy than histologic grade and reveal biological features that may have therapeutic implications, such as immune signatures or proliferative pathway activation.

### 3.3 Clinical implications

Methylation profiling increasingly informs real-world clinical decision-making. It aids in predicting recurrence risk following gross total resection, identifies histologically benign-appearing tumors with aggressive molecular features, guides the need for adjuvant radiotherapy, and assists in selecting patients for molecularly tailored clinical trials. For recurrent, atypical, and morphologically ambiguous meningiomas, methylation profiling is rapidly becoming an essential diagnostic complement.

## 4 High-risk prognostic molecular markers

Advances in molecular diagnostics have identified specific genetic and epigenetic alterations that independently predict prognosis in meningiomas (15).

**Figure 3:**
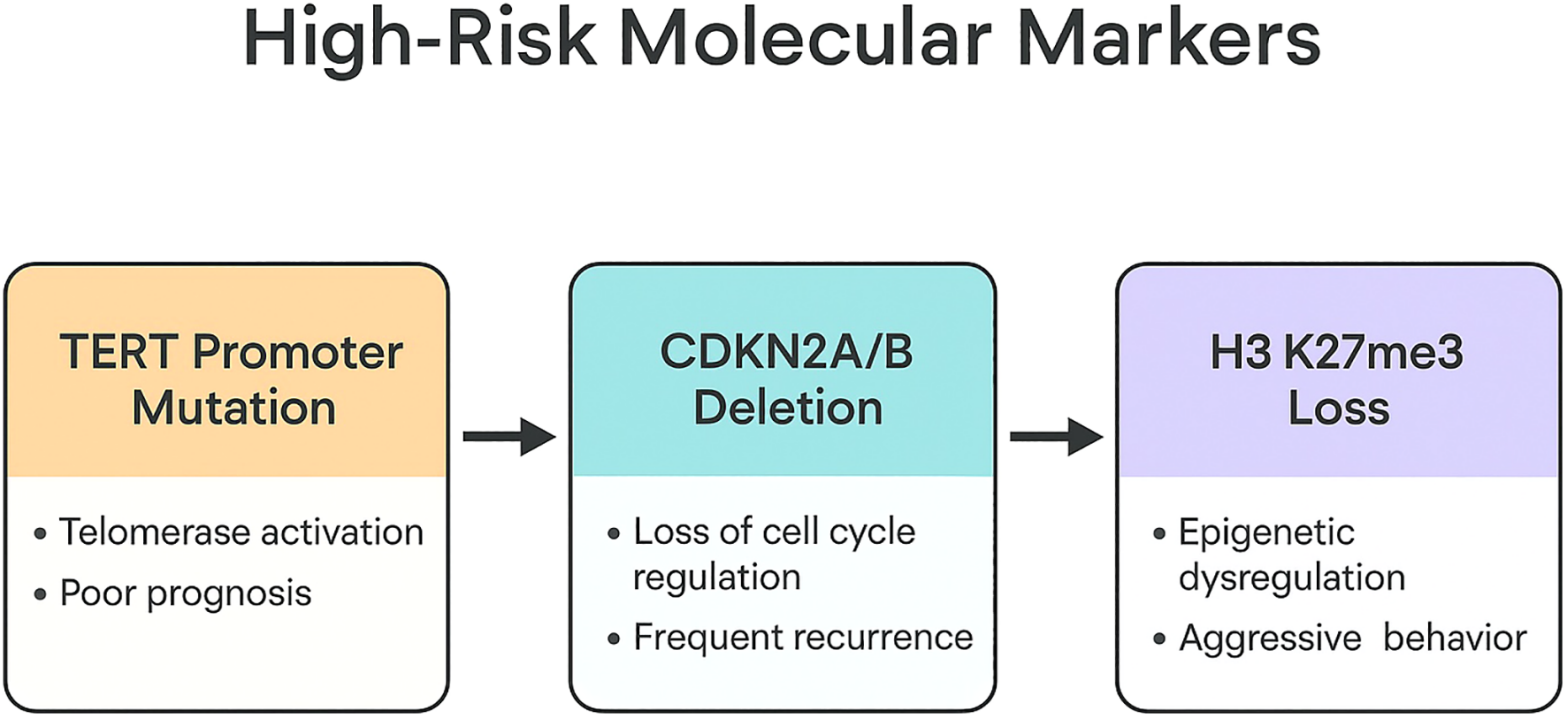
High-risk molecular markers associated with aggressive behavior including TERT promoter mutation, CDKN2A/B deletion, and H3K27me3 loss.

Mutations in the TERT promoter (TERTp), particularly C228T and C250T substitutions, occur in approximately 3% to 5% of meningiomas (**Figure 3** summarizes high-risk molecular markers that independently predict recurrence and survival) and are strongly associated with early recurrence, malignant progression, and significantly reduced survival. These mutations are now regarded as an adverse prognostic feature sufficiently powerful to define WHO grade 3 in some contexts, even when histology is low-grade.

Homozygous deletions of CDKN2A/B, which encode critical cell-cycle regulators, represent another robust predictor of poor outcome and have been incorporated into the WHO classification as a molecular marker of grade 3 meningioma. These deletions frequently co-occur with NF2 alterations and high chromosomal instability, reinforcing their association with aggressive behavior.

Epigenetic markers such as loss of H3K27me3, a histone modification associated with transcriptional repression, also correlate with poor prognosis, particularly among atypical meningiomas. Tumors lacking H3K27me3 expression demonstrate significantly shorter recurrence-free survival. This readily assessable immunohistochemical marker enhances risk stratification when combined with molecular and histologic data.

Global copy-number burden further contributes to prognostication. Losses involving chromosomes 1p, 6q, 10q, and 14q are associated with aggressive behavior, and tumors with high copy-number variation show markedly increased recurrence rates (15).

## 5 Liquid biopsy and circulating biomarkers

**Figure 4:**
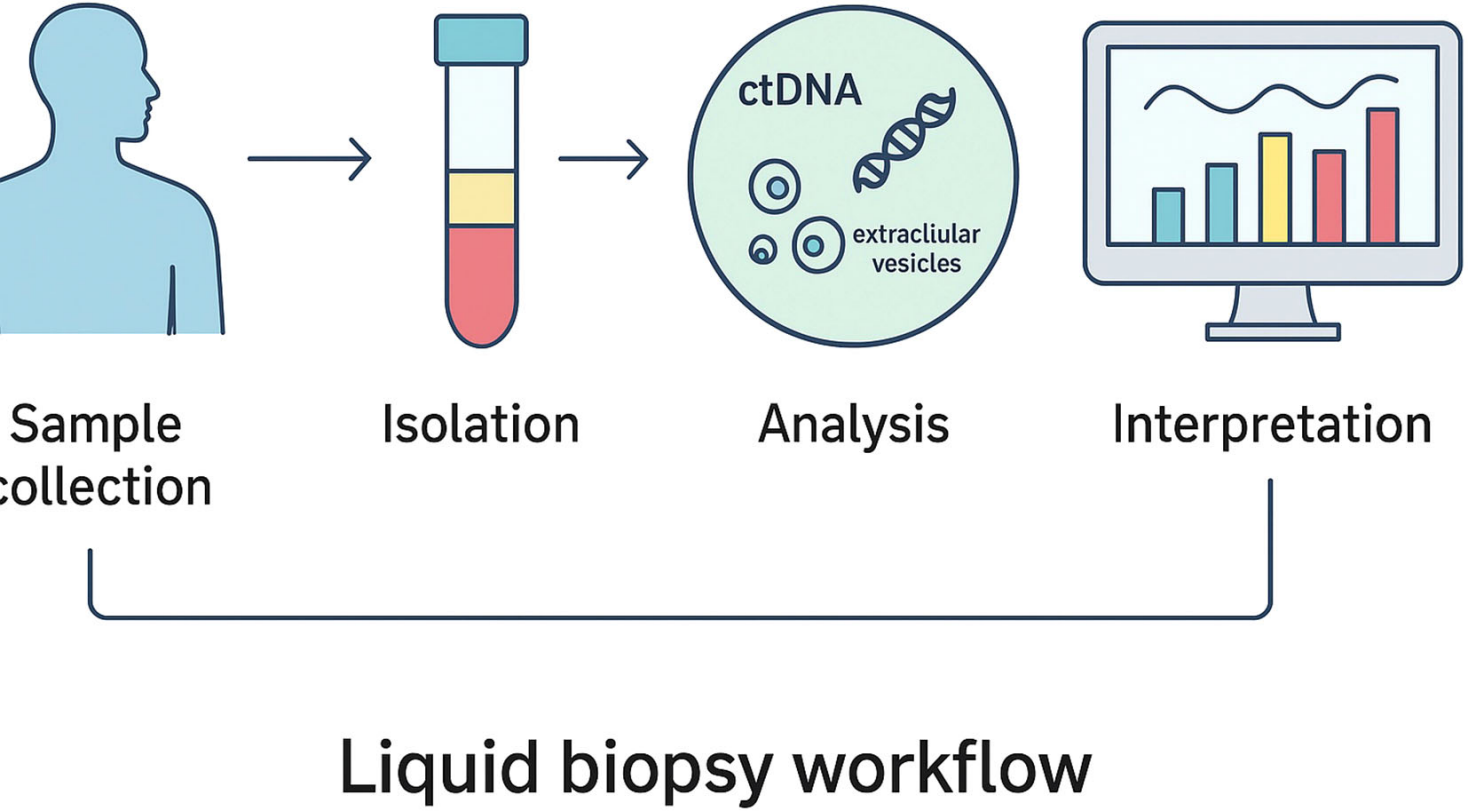
Liquid biopsy workflow illustrating sample collection, circulating tumor component isolation, molecular analysis, and clinical interpretation.

Liquid biopsy has emerged as a promising adjunct to conventional imaging and tissue-based diagnostics in meningioma. Circulating tumor DNA (ctDNA) can be detected in plasma and carries tumor-specific genetic and epigenetic information (**Figure 4** presents the workflow of liquid biopsy and its potential clinical applications). Several studies have demonstrated that meningioma-specific methylation signatures can be identified in plasma-derived cell-free DNA (cfDNA), providing a non-invasive approach to tumor detection and molecular classification (16). In addition, liquid biopsy enables real-time monitoring of tumor dynamics, with ctDNA levels increasing in parallel with tumor progression and, in some cases, allowing earlier detection of recurrence compared with conventional MRI imaging (17). Serial plasma sampling also permits assessment of molecular evolution over time, including the acquisition of adverse alterations such as TERT promoter mutations during disease progression (18).

Beyond ctDNA, circulating microRNAs—including miR-21 and miR-34a—have shown differential expression between benign and aggressive meningiomas, suggesting potential roles as minimally invasive biomarkers (19). Proteomic markers such as PD-L1, VEGF-A, and proinflammatory cytokines have likewise been explored for their prognostic and therapeutic relevance (20). Despite these encouraging findings, important limitations remain. Sensitivity of liquid biopsy is reduced in low-volume disease and may vary according to tumor size, location, and vascularity. Furthermore, most available evidence is derived from retrospective or exploratory studies, underscoring the need for prospective validation in larger, clinically annotated cohorts before routine clinical implementation.

## 6 Therapeutic implications and targeted treatment

The characterization of specific molecular subgroups has provided a foundation for targeted therapeutic strategies in meningioma, although effective systemic treatments remain limited.

Tumors harboring SMO mutations represent an actionable subgroup for Hedgehog pathway inhibitors. Early-phase clinical trials of agents such as vismodegib and sonidegib have demonstrated modest disease stabilization in selected patients, underscoring the need for molecularly enriched cohorts (7).

**Figure 5:**
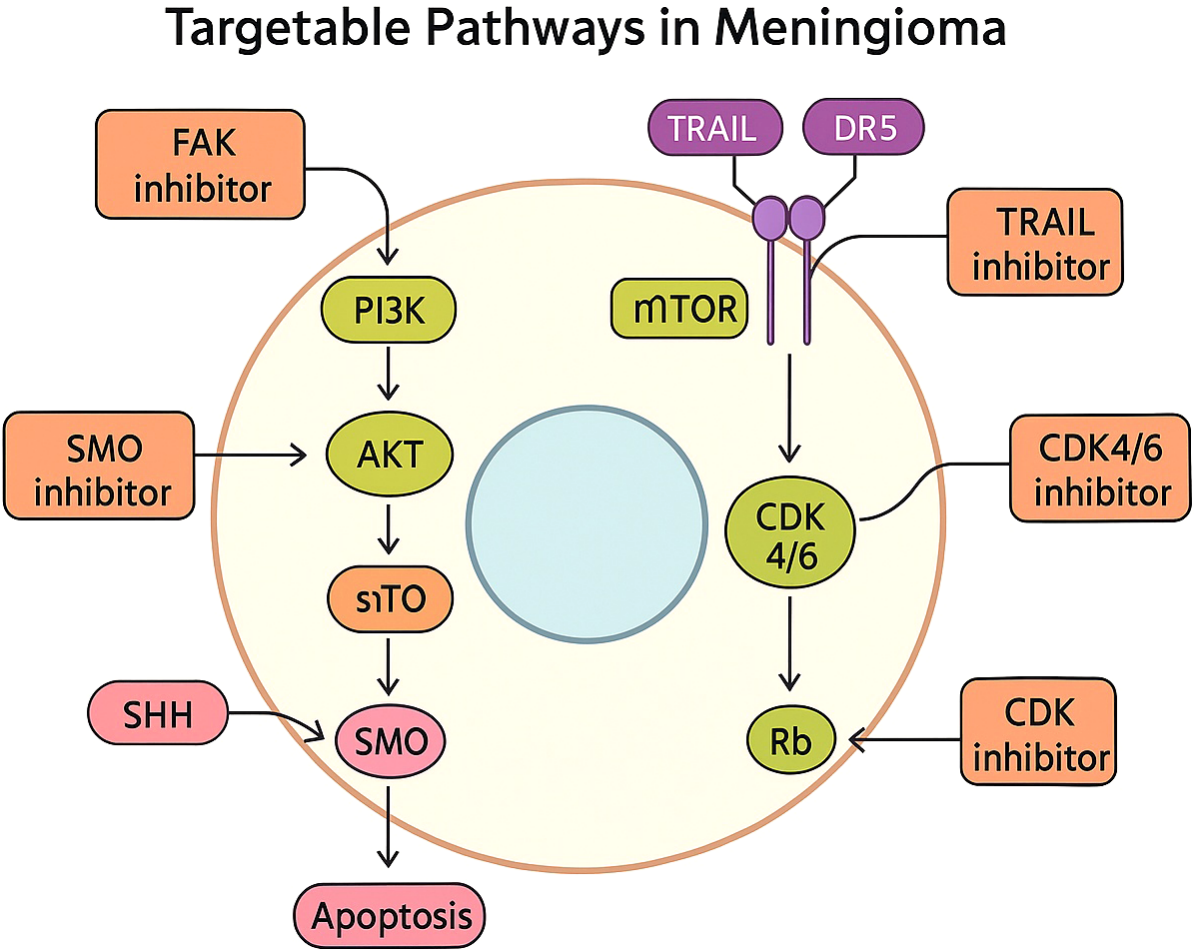
Targetable molecular signaling pathways in meningioma including PI3K/AKT/mTOR, Hedgehog, FAK, and cell-cycle pathways.

Alterations in the PI3K/AKT/mTOR pathway, including AKT1 and PIK3CA mutations, justify the investigation of pathway inhibitors (**Figure 5** highlights actionable signaling pathways and corresponding targeted therapies). AKT inhibitors such as capivasertib and PI3K inhibitors such as alpelisib are being evaluated in molecularly selected patients. The mTOR inhibitor everolimus has shown mixed results, particularly when combined with somatostatin analogues like octreotide (11).

NF2-mutant meningiomas may benefit from FAK inhibitors, given the central role of FAK signaling in Merlin-deficient tumors. Clinical trials of GSK2256098 have shown early evidence of disease stabilization in this subgroup (21).

In tumors characterized by CDKN2A/B deletion or cell-cycle dysregulation, CDK4/6 inhibitors such as abemaciclib have demonstrated promising early activity. Epigenetically dysregulated meningiomas, particularly those harboring SWI/SNF complex alterations such as ARID1A or SMARCB1 mutations, may be sensitive to EZH2 inhibitors, with early-phase trials of tazemetostat supporting further exploration (22).

Although meningiomas typically exhibit low mutational burden, some tumors show immune-rich microenvironments. Immune checkpoint inhibitors such as pembrolizumab and nivolumab have been tested in recurrent high-grade meningioma, demonstrating limited but noteworthy activity (23).

## 7 Clinical integration of molecular profiling

The integration of molecular profiling into clinical practice has significantly improved diagnostic precision, prognostication, and treatment planning. Tumors with unique molecular signatures—such as secretory meningiomas characterized by combined KLF4 and TRAF7 mutations, clear cell meningiomas driven by SMARCE1 loss, or BAP1-inactivated rhabdoid tumors—can now be accurately classified based on their genetic alterations, even when histology is ambiguous. These molecular insights aid pathologists and clinicians in refining diagnoses that may influence subsequent management.

Prognostically, markers such as TERT promoter mutation, CDKN2A/B deletion, H3K27me3 loss, and methylation class are increasingly used to guide decisions regarding adjuvant radiotherapy and intensity of surveillance. Tumors classified within aggressive methylation groups or harboring high-risk molecular features may warrant closer imaging follow-up or early postoperative radiotherapy, even when histology suggests low-grade disease.

Perhaps most importantly, molecular profiling facilitates personalized treatment strategies through selection of patients for clinical trials evaluating targeted agents or immunotherapies. The identification of actionable mutations such as SMO, AKT1, or PIK3CA allows patients to be enrolled in genotype-directed therapeutic studies, representing a critical step toward precision medicine in meningioma.

## 8 Limitations and future directions

Despite significant progress, challenges remain:

- Limited access to methylation profiling in low-resource settings.
- Lack of highly effective systemic therapies; most targeted treatments result in disease stabilization, not regression.
- Intratumoral heterogeneity complicates single-biopsy interpretation.
- Need for standardized molecular reporting systems integrating histology, CNV, methylation, and mutations.
- Requirements for prospective multi-omic trials to validate classifiers and therapeutic responses.
- Future research will likely emphasize combined radiotherapy–molecular inhibitor strategies, liquid biopsy–guided monitoring, and deeper insights into spatial heterogeneity and tumor microenvironment.

Addressing these challenges will require integration of emerging biological insights, minimally invasive molecular diagnostics, advanced computational tools, and collaborative data infrastructures.

### 8.1 Emerging molecular insights in WHO grade 3 meningiomas

Recent work has further refined the biological understanding of grade 3 meningiomas, highlighting the central role of TERT promoter mutations, CDKN2A/B homozygous deletions, high copy-number burden, and proliferative methylation-defined subgroups in driving aggressive behavior (24). These tumors exhibit marked genomic instability, dysregulated cell-cycle control, and enrichment of DNA repair and proliferation pathways. Recognition of these molecular characteristics supports incorporation of molecular risk factors into treatment algorithms, including consideration of early adjuvant radiotherapy, intensified surveillance schedules, and prioritization for enrollment in molecularly targeted clinical trials.

### 8.2 Blood-based molecular profiling for surgical decision-making

Liquid biopsy approaches are being explored as adjuncts to tissue-based diagnostics in meningioma. Circulating tumor DNA (ctDNA) and tumor-specific methylation signatures detectable in plasma offer a non-invasive means of tumor classification and disease monitoring (16). Although sensitivity remains limited in low-volume disease, ongoing improvements in assay sensitivity raise the possibility that blood-based molecular profiling may assist in preoperative risk stratification, particularly in tumors located in surgically challenging regions. Integration of plasma-derived molecular data with imaging and clinical parameters may eventually help guide the extent of surgical resection, inform adjuvant therapy decisions, and tailor postoperative surveillance strategies (17).

### 8.3 Artificial intelligence and computational modeling

Emerging artificial intelligence (AI)–driven modeling approaches are increasingly being integrated with molecular profiling in meningioma research. Machine learning frameworks combining radiomic features, genomic alterations, DNA methylation patterns, and clinical variables have demonstrated potential for improving risk stratification and recurrence prediction. Integrative AI platforms can identify imaging–molecular correlations that are not readily apparent through conventional analysis, enabling non-invasive inference of tumor biology and facilitating longitudinal disease tracking (25). As these tools mature, AI-assisted systems may support personalized surveillance strategies and treatment planning by dynamically integrating molecular, radiologic, and clinical data.

### 8.4 Need for centralized molecular data repositories

A key limitation in advancing meningioma molecular research is fragmentation of datasets across institutions. Establishment of centralized, multi-institutional repositories integrating genomic, epigenetic, radiologic, and clinical outcome data would substantially accelerate validation of molecular classifiers and therapeutic targets. Such initiatives would require standardized biospecimen processing protocols, harmonized bioinformatics pipelines, secure data-sharing infrastructure, and robust ethical governance frameworks. Centralized repositories could facilitate large-scale validation studies, improve reproducibility, and enable AI-driven predictive modeling, thereby enhancing the translational impact of molecular profiling.

## 9 Conclusion

Meningiomas, once viewed as primarily histologically defined tumors, are now recognized as molecularly diverse neoplasms driven by complex genomic, epigenomic, and transcriptomic alterations. Advances in molecular profiling—including driver mutation characterization, DNA methylation classification, and identification of high-risk prognostic markers—have dramatically improved diagnostic accuracy and risk stratification. Emerging targeted therapies and liquid biopsy technologies promise to reshape management strategies, particularly for recurrent or aggressive tumors. As molecular diagnostics become increasingly integrated into clinical practice, tailored therapeutic approaches and improved patient outcomes are within reach.
